# Withdrawn: Circ‐SOX4 promotes non‐small cell lung cancer progression by activating the Wnt/β‐catenin pathway

**DOI:** 10.1002/1878-0261.12656

**Published:** 2020-11-15

## Abstract

The above article, published online on 28 February 2020 in Wiley Online Library (wileyonlinelibrary.com) as an Accepted Article (https://doi.org/10.1002/1878‐0261.12656), has been withdrawn at the authors' request, and in agreement between the authors, the journal Editors in Chief J. E. Celis and K. Ryan, and John Wiley & Sons Ltd. The withdrawal has been agreed because the authors were unable to obtain approval to publish the final version from all authors.

